# An osmolarity dependent mechanism partially ameliorates retinal cysts and rescues cone function in a mouse model of X-linked retinoschisis

**DOI:** 10.3389/fmed.2024.1302119

**Published:** 2024-10-15

**Authors:** Ella J. Gehrke, Jacob Thompson, Emily Kalmanek, Sarah T. Stanley, Joseph Laird, Sajag Bhattarai, Brianna Lobeck, Sara Mayer, Angela Mahoney, Salma Hassan, Ying Hsu, Arlene Drack

**Affiliations:** ^1^Department of Ophthalmology and Visual Sciences, Institute for Vision Research, University of Iowa, Iowa City, IA, United States; ^2^Department of Epidemiology, College of Public Health, University of Iowa, Iowa City, IA, United States; ^3^Department of Biochemistry and Molecular Biology, University of Iowa, Iowa City, IA, United States; ^4^Biomedical Science-Cell and Developmental Biology Graduate Program, University of Iowa, Iowa City, IA, United States; ^5^Department of Anatomy and Cell Biology, University of Iowa, Iowa City, IA, United States; ^6^Interdisciplinary Graduate Program in Genetics, University of Iowa, Iowa City, IA, United States; ^7^Department of Pediatrics and Interdisciplinary Genetics Program, University of Iowa, Iowa City, IA, United States

**Keywords:** X-linked retinoschisis, retinoschisin, disease mechanisms, gene therapy, osmolarity, hypertonic, electroretinogram, subretinal

## Abstract

**Introduction:**

X-linked retinoschisis (XLRS) is a vitreoretinal dystrophy caused by *RS1* gene mutations which disrupt retinoschisin-1 (RS1) function. Vital for retinal architecture, the absence of functional RS1 leads to the development of intraretinal cysts. Intravitreal injection of a gene therapy for treating XLRS caused ocular inflammation in high dose groups in a phase I/II clinical trial. This study investigates a low dose subretinal gene therapy in *Rs1* knockout (*Rs1*-KO) mice compared to injection of buffer alone. Observation of an unexpected therapeutic effect following the subretinal injection of the hypertonic buffer led to novel findings in XLRS.

**Methods:**

*Rs1*-KO mice were subretinally injected with an AAV2/4 vector (*n* = 10) containing the *RS1* gene driven by an Ef1α promoter, a hypertonic buffer (*n* = 15) (180 mM NaCl 0.001% F68/PBS (pH 7.4)), or isotonic buffer (*n* = 7) (155.2 mM NaCl 0.001% F68/PBS, pH 7.0). A sham puncture group was also included (*n* = 6). Endpoints included electroretinogram (ERG), optical coherence tomography (OCT), a visually guided swim assay (VGSA), and immunohistochemistry.

**Results:**

Unexpectedly, hypertonic buffer-injected eyes had reduced cyst severity at 1-month post-injection (MPI) (*p* < 0.0001), higher amplitudes in cone-dominant ERGs persisting to 5 MPI (5 Hz flicker; *p* < 0.0001; 3.0 flash; *p* = 0.0033) and a trend for improved navigational vision in the light compared to untreated *Rs1*-KO eyes. To investigate the role of tonicity on this effect, an isotonic buffer-injected cohort was created (155.2 mM NaCl 0.001% F68/PBS, pH 7.0) (*n* = 7). Surprisingly, hypertonic buffer-injected eyes exhibited a greater reduction in cyst severity and demonstrated improved cone-dominant ERG metrics over isotonic buffer-injected and sham puncture eyes. An immunohistochemistry assay demonstrated greater cone density in hypertonic buffer-injected eyes than untreated *Rs1*-KO eyes at 5–6 MPI (*p* = 0.0198), suggesting a possible cone preservation mechanism. Moreover, our findings reveal a negative correlation between the peak severity of cysts and long-term ERG amplitudes in cone-dominant pathways, implying that effectively managing cysts could yield enduring benefits for cone function.

**Discussion/conclusion:**

This study presents evidence that cyst resolution can be triggered through an osmolarity-dependent pathway, and early cyst resolution has long-term effects on cone signaling and survival, offering potential insights for the development of novel treatments for XLRS patients.

## 1 Introduction

Juvenile X-linked retinoschisis (XLRS), a vitreoretinal disorder, is the leading cause of macular dystrophy in young males, with a global prevalence of up to 1 in 5,000 individuals (1). The pathology of the disease involves an X-linked recessive mutation in the retinoschisin-1 (*RS1*) gene located at Xp22.1–p22.3 (1). Mutations in *RS1*, of which there are greater than 200 known, impair production of the 224 amino acid protein, retinoschisin-1 (RS1) (1). RS1 is primarily secreted by photoreceptors and bipolar cells, and influences cell–cell interactions through a ubiquitous discoidin domain, facilitating photoreceptor-bipolar cell signaling, and stabilizing the extracellular environment of the retina (2, 3). In the absence of functional RS1, the laminar architecture of the retina becomes disrupted, causing retinal layer separation, known as schisis, and the formation of retinal cysts. Cysts, a hallmark of XLRS, spatially divide the inner nuclear and outer plexiform layers, separating the bipolar cells and photoreceptors. This separation impedes electrical transduction in the retina, leading to a characteristic waveform in electroretinography (ERG) termed an electronegative ERG, in which the ascending b-wave does not exceed the baseline.

From a clinical perspective, almost all patients with XLRS experience foveal involvement, impairing central vision (4, 5). Comparatively, around 50 percent of patients also exhibit peripheral involvement (2, 4). The progressive impairment of central vision due to macular atrophy can result in substantial functional vision loss. Additionally, the development of schisis significantly increases the risk of vitreous hemorrhage and full thickness retinal detachment, especially following even mild ocular trauma (4). Strategies to manage cyst severity are needed.

Currently, carbonic anhydrase II inhibitors (CAIs) serve as the primary medical therapy available for patients with XLRS. The proposed mechanism of CAIs involves inhibition of membrane-bound carbonic anhydrase (CA) in the retinal pigment epithelium (6). Fluid transport from the subretinal space into the choroid is enhanced experimentally following introduction of a carbonic anhydrase inhibitor (CAI), acetazolamide, suggesting CA is involved in maintaining fluid homeostasis of the retina (6). However, despite their efficacy in reducing intraretinal cyst formation in some patients, systemic CAIs are associated with well-known systemic side effects affecting the endocrine, gastrointestinal, cardiovascular, and central nervous systems, while there is often poor compliance with topical CAIs, especially in children (7–9).

While CAIs have shown short term benefits including mild decrease in central macular thickness, reduction of intraretinal cyst formation, and improved vision, it is not known whether they prevent the long-term progression of XLRS (8). Additionally, several studies have reported limited effects on retinal morphology or functional benefit, leading to inconsistent use of CAIs across different treatment centers (5, 10).

Sudden discontinuation of topical brinzolamide, a commonly used CAI in clinical practice, can lead to increased cyst formation and elevated intraocular pressure during periods of drug holiday (8, 9). There are currently no studies demonstrating long-term efficacy or safety, and CAIs are at best a treatment with variable efficacy, not a cure.

Given the lack of effective long-term therapy, ongoing efforts in gene therapy have emerged as a potential avenue for treatment. A Phase I/IIa trial was conducted on human subjects which involved a single intravitreal injection of an AAV8 vector carrying the *RS1* gene (11). This safety study was introduced after good success in a mouse model; however, in human subjects, several ocular inflammatory outcomes were recorded (11). An additional human study utilizing an AAV2 vector to deliver intravitreal *RS1* also reported inflammatory outcomes (12).

Compared to intravitreal injections delivering equivalent doses of AAV, subretinal injections elicited lower levels of humoral immune reactions both in mice and in primates (13). In our study, we investigate whether a subretinal injection of low-dose adeno-associated virus, AAV2/4-EF1α-*RS1*, can rescue the retinal phenotype with reduced risk of vector toxicity and ocular inflammation. In this study, we subretinally administered a low dose AAV2/4-EF1α-*RS1* gene therapy to *Rs1*-KO mice, a model for XLRS. To account for the potential effect of the subretinal injection procedure, a control group receiving a subretinal injection of a hypertonic buffer, similar in osmolarity to the AAV storage buffer, was included.

We hypothesized that subretinal injections of low dose AAV2/4-EF1α-*RS1* could correct the retinal phenotype in the *Rs1*-KO mouse detectable by electroretinography (ERG), optical coherence tomography (OCT), and a visually guided swim assay (VGSA). However, our experiments revealed an unexpected therapeutic effect of the buffer, including cyst reduction accompanied by long-term ERG improvement, constituting a novel finding in XLRS.

## 2 Methods

### 2.1 Study design

Natural history, as well as gene therapy efficacy and effect of subretinal buffer injections, were evaluated in a mouse model of juvenile X-linked retinoschisis (*Rs1*-KO). The natural history of this *Rs1*-KO mouse model has been previously reported, including the variable expressivity of the phenotype (14). We examined treatment-naïve eyes from postnatal day (p) 15- to 12-months of age. OCT images were analyzed for both outer nuclear layer (ONL) thickness and cyst severity.

To study the effect of gene therapy, eyes treated with a subretinal injection of gene therapy vector (AAV2/4-EF1α-*RS1*) (*n* = 10) were compared to hypertonic buffer-injected eyes (*n* = 15) and treatment-naïve eyes (*n* = 30). The hypertonic buffer-injected cohort was initially included to account for the high salt content in the solution where the vector is produced and stored, as part of the standard formulation to prevent viral clumping. Injections alternated between the left and right eyes to avoid systematic bias. The selection of an AAV2/4 vector was based on a previously demonstrated tropism for all layers of the retina, including the photoreceptor and bipolar cell layers, the primary secreters of RS1 (15).

Injections were performed between p24 and p31. Outcome measures include ERG, OCT, VGSA, and immunohistochemistry. ERG was performed at 1-, 2-, 3-, and 5-months post injection (MPI) to assess retinal function. OCT was performed at 2- and 3-weeks post-injection (WPI), then 1-, 2-, 3-, and 5 MPI to assess cyst severity and retinal structure. A visually guided swim assay was performed at 5 to 7-months of age to assess functional vision. The eyes were collected and fixed at 6 MPI for immunohistochemistry. For the purpose of ERG and OCT experiments, one eye is considered a biological data point. For the purpose of the VGSA, each data point is representative of the averaged time-to-platform of a mouse for 20 different trials using randomly selected platform positions. Mice were treated in one eye with either vector or hypertonic buffer. Mice treated with gene therapy in one eye and hypertonic buffer in the fellow eye were excluded from the VGSA. Both male (*Rs1* hemizygous mutants) and female knockout mice (*Rs1* homozygous mutants) were used in this study.

To investigate the effect of the buffer, the subretinal injection of the hypertonic buffer was compared to the subretinal injection of an isotonic buffer and a sham puncture injection group that included a mechanical puncture of the sclera without deposition of a subretinal buffer fluid. Endpoints included ERG and OCT, and the same timelines as stated above were used in this group.

### 2.2 Animal husbandry and ethics statement

This study was performed in accordance with the recommendations set forth by the National Institute of Health in the Guide for the Care and Use of Laboratory Animals. Animals were handled in accordance with the approved Institutional Animal Care and Use Committee (IACUC) protocol #4031421 at the University of Iowa. The *Rs1*-KO C57Bl/6J mouse model was kindly supplied by Paul Sieving, M.D., Ph.D. at the National Eye Institute. Generation of the *Rs1*-KO mouse model was previously described (16). This mouse model contains a deletion of exon 1 and a 1,630 bp fragment of intron 1 on the *Rs1* gene (16).

Animals were housed according to the IACUC recommendations. Humane endpoints were observed, and the method of euthanasia used was carbon dioxide inhalation followed by cervical dislocation.

### 2.3 Statistical analysis

Analysis was performed using GraphPad PRISM 10.0 (GraphPad Software, Inc., San Diego, California, United States). Two-way ANOVA was performed for ERG and OCT metrics and followed by multiple comparisons (Tukey’s test, non-parametric). One-way ANOVA was performed for VGSA and cone density followed by multiple comparisons (Tukey’s test, non-parametric). Simple linear regression was used for correlation of peak cyst area vs. 5 MPI ERG amplitudes.

### 2.4 Genotyping information

Genotyping was performed using Taq Polymerase (New England biolabs, Ipswich, MA) using the primers listed in Table 1. The PLA2 primer was previously described (16). A 10 microliters (μL) reaction volume was used containing 2 μL of DNA (approximately 25 μg/μL), 3.7 μL of ultrapure water, 1 μL of 10X Buffer/Mg++, 1 μL of 20 mM primer mix (Table 1), 0.2 μL of 50 mM dNTPs, 0.1 μL of rTaq, and 2 μL of 5X Betaine. Cycling conditions are as follows; initial denaturing at 94°C for 30 s, annealing at 57°C for 30 s and extension at 72°C for 30 s, with a final extension step at 72°C for 4 min. This produces a 516-base pair (bp) wild-type band and a 300 bp knockout band.

**Table 1:** Primers.

*RS1* promoter F2 33%	5′-TAGGGGCCCACATCTTCCAAC-3′
PLA2 33%	5′-GTTCTTCGGACGCCTCGTCAACAC-3′
RSWT2-R 33%	5′-GTGACAAAGAGCCACACAACAGTGACC-3′

### 2.5 AAV packaging

The human *RS1* gene was cloned into a shuttle plasmid pFBAAV and provided by the Viral Vector Core at the University of Iowa. The bacterial backbone is from Invitrogen’s pFastBac system. The shuttle plasmid consists of the pFBAAV backbone, an EF1α promoter, and a bovine growth hormone polyadenylation signal. The plasmid was packaged into an AAV2/4 capsid by the Budd Tucker Laboratory at the University of Iowa. The AAV was formulated at 2 × 10^9^ viral genomes (vg)/mL titer, and the composition of the storage buffer is listed in Table 2. Titering was performed using digital droplet PCR.

**Table 2:** Buffer solutions.

AAV storage buffer	180 mM NaCl 10 mM Na_3_PO_4_ UltraPure water pH 7.2–7.4
Hypertonic injection buffer	0.001% Gibco Pluronic F-68 Gibco dPBS (−/−) (Catalog #10010) (contains 155.2 mM NaCl) Additional 24.8 mM NaCl (for 180 mM NaCl total) pH 7.4
Isotonic injection buffer	0.001% Gibco Pluronic F-68 Gibco dPBS (−/−) (Catalog #10010) (contains 155.2 mM NaCl) pH 7.4

### 2.6 Subretinal injection

Mice were anesthetized using a ketamine/xylazine mixture (87.5 mg/kg ketamine, 12.5 mg/kg xylazine). A tropicamide ophthalmic solution of 1% was applied 3 min prior to injection to dilate the eyes. Proparacaine was applied as a topical anesthetic and 10% povidone-iodine was applied as a topical antiseptic. A partial thickness puncture was made just posterior to the limbus through the sclera with a 30-G sharp needle, then a 33-G blunt Hamilton needle was inserted and positioned between the RPE and the retina. Two μL of either gene therapy vector at a concentration of 2 × 10^9^ vg/mL, a hypertonic buffer, or an isotonic buffer was injected into the subretinal space (formulation available in Table 2). The injection of a solution subretinally creates a separation of the retina and the RPE known as a subretinal bleb. This bleb was assessed by the injector as seen through the operating microscope and in the rare cases with no visible bleb or a large vitreous hemorrhage, the mouse was excluded. For the sham injection puncture experiment, the same sharp needle was used to make an entry into the eye, then the blunt Hamilton needle was introduced subretinally however no liquid was delivered. Mice with retinal detachments that persisted until the first OCT time point were excluded. The mouse was then given topical ophthalmic ointment consisting of neomycin, polymyxin B sulfates, bacitracin zinc, and hydrocortisone. Mice were injected with atipamezole to aid in recovery from the anesthetic.

### 2.7 Electroretinogram

Dark adaptation occurred overnight, and ERGs are conducted in the morning to control for potential diurnal variations. Mice were anesthetized using a ketamine/xylazine mixture (87.5 mg/kg ketamine, 12.5 mg/kg xylazine). A tropicamide ophthalmic solution of 1% was applied to the eyes for pupil dilation. A 2.5% Hypromellose solution (Akron, Lake Forest, Illinois) was applied to maintain corneal hydration during testing. Testing was performed on the Diagnosys Celeris with integrated heated platform (Diagnosys, Lowell, MA). Electrodes were placed on the cornea of bilateral eyes, and impedance was kept below 10 kΩ. Eyes were exposed to various light intensities in a modified ISCEV protocol (17). To evaluate rod electrical function in treated and untreated *Rs1*-KO mice, animals were dark-adapted (DA) overnight to isolate the rod response, and eyes were subjected to 15 flashes of 0.01 cd·s/m^2^ light (0.01 dim flash) then 15 flashes of dark-adapted 3.0 cd·s/m^2^ standard combined response (3.0 SCR). To evaluate cone electrical function in treated and untreated *Rs1*-KO mice, eyes were light-adapted (LA) for 10 min to bleach the responses from rods, and subjected to 15 flashes of 3.0 cd·s/m^2^ light (3.0 flash) followed by 20 flashes of a 5 Hz flickering light of 3.0 cd·s/m^2^ (5 Hz flicker), which is more reproducible in mice than the standard light-adapted 30 Hz flicker in humans (18). Multiple comparisons were performed between every group at each time point. Non-significant comparisons were not graphed.

### 2.8 Optical coherence tomography

**Figure 1:**
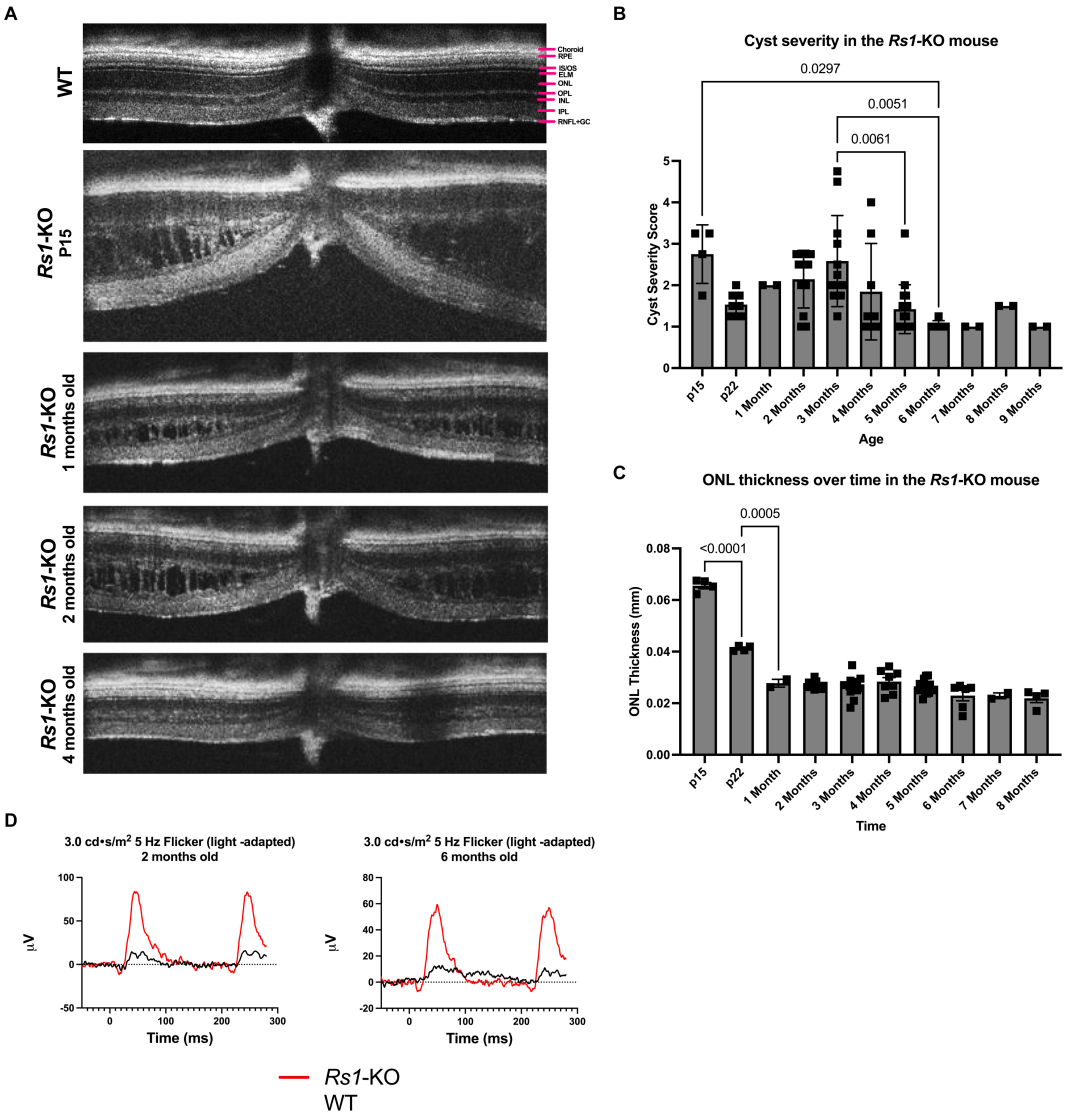
**(A–D)** Layers of the WT mouse retina is represented as follows; retinal nerve fiber layer and ganglion cell layer (RNFL + GC), inner plexiform layer (IPL), inner nuclear layer (INL), outer plexiform layer (OPL), outer nuclear layer (ONL), external limiting membrane (ELM), photoreceptor inner and outer segments (IS/OS), retinal pigment epithelium (RPE), and choroid **(A)**. Natural history of disease in the *Rs1*-KO mouse compared to WT BL/6 mouse. OCT images were collected from p15 to 9-months of age in *Rs1*-KO mice. Representative OCT images demonstrate retinoschisis in *Rs1*-KO mice at p15, 1-, 2- and 4-months old, compared to a WT mouse without retinoschisis **(A)**. Cyst severity and ONL thickness were measured at each age. To calculate cyst severity **(B)**, cyst height was measured at 4 different positions in an eye and translated to a scoring scale of 1 to 6, with 1 representing a cyst height of 0. ONL thickness **(C)** was measured overtime. ERGs of *Rs1*-KO mice and WT mice were collected at 2- and 6-months of age. Representative waveforms are demonstrated **(D)**. As early as 2-months of age, *Rs1*-KO mice show reduced ERG b-wave amplitudes and delayed latency of the b-wave peak, compared to the robust WT waveforms.

Mice were anesthetized using a ketamine/xylazine mixture (87.5 mg/kg ketamine, 12.5 mg/kg xylazine), and tropicamide ophthalmic solution 1% was applied for pupil dilation. 1% carboxymethylcellulose was applied to maintain corneal hydration during testing. OCTs were conducted in the afternoon to control for potential diurnal variations. Cyst severity in the natural history data (Figure 1) was determined via a modified scoring protocol from previously described literature (19, 20). Briefly, measurements of cyst height were taken at four points equidistant, 500 μm from the optic nerve, two on the superior–inferior line bisecting the optic nerve, and two on the nasal-temporal line bisecting the optic nerve. Measurements were then translated to a scoring scale as previously described, in which a score of 1 was assigned to zero cyst height and a score of 6 was assigned to cyst height >100 μm. The score was averaged across all four points (20).

For all other cyst severity studies, volumetric scans were taken centering on the optic nerve along with nasal and temporal scans. Cyst severity was determined via manual segmentation of cyst area using the ImageJ software. Center scans, those which best bisect the optic nerve, were quantified by manually segmenting individual cyst cavities and recording the total area of cysts in the scan. Similar quantifications were performed on nasal-temporal slices located approximately 500 μm superior and inferior of the center scan, and area was reported as a sum of these measurements. Multiple comparisons were performed between every group at each time point. Non-significant comparisons were not graphed.

### 2.9 Visually guided swim assay

AAV2/4-EF1α-*RS1* vector-treated mice and mice injected with the hypertonic buffer underwent a VGSA performed in light and dark conditions to assess functional vision at approximately 5 to 7-months of age compared to treatment-naïve controls. The VGSA has been described in detail elsewhere (21). In brief, the mice undergo four training days in the light, four testing days in the light, two training days in the dark, and four testing days in the dark, and the performance of each mouse to 5 different platform locations were measured during each training or testing day. For each trial, the mice were placed into a plastic pool and were expected to find a platform. Five of the eight possible platform positions are randomly assigned each day with all platforms being used approximately the same number of times. The maximum time to platform (TTP) was set at 60 s for each trial to prevent fatigue and undue stress. After the mouse had completed the trial or after 60 s of searching, the mouse was removed from the pool, dried, and placed in its cage until the next trial. Mice were excluded in rare cases where they could not be motivated to participate and search for the platform. Data from all trials was excluded if a mouse had a swim time greater than one standard deviation from the mean of the group and consistent floating occurred, such as more than three episodes of floating per trial or more than three corrections required per trial. Corrections for floating include snapping fingers or touching the mouse’s tail.

### 2.10 Immunohistochemistry

Eyes were enucleated and a small puncture was made through the cornea with a 27-G needle. Eyes were placed in 4% paraformaldehyde for 24 h and transferred to PBS for 24 h. After fixation, eyes were embedded in Tissue-Tek O.C.T. compound (VWR, Batavia, IL) and frozen in a bath of 2-methylbutane cooled with liquid nitrogen. Eyes were sectioned on the superior–inferior axis at a thickness of 10 μm using a Cryostat microtome and stored at-80°C for future use.

For immunohistochemistry, sections were permeabilized using 0.3% Triton X-100 in PBS (buffered at pH 7.4) for 10 min at room temperature, blocked with a blocking buffer containing 5% BSA, 5% normal goat serum, and 0.05% Triton X-100 in PBS for 1 h at room temperature, and then incubated with primary antibodies or biotinylated-peanut agglutinin (biotinylated-PNA) in a dilution buffer containing 5% BSA, 1% normal goat serum, and 0.05% Triton X-100 in PBS at 4°C overnight. The following day, samples were washed in 1xPBS three times before incubation with secondary antibodies or streptavidin Alexa Fluor-568 conjugate at room temperature for 1 h. After washing in 1xPBS an additional three times, samples were mounted with VectaShield mounting medium with DAPI (Vector Laboratories, Burlingame, CA). Images were taken using a fluorescence microscope and color channels were overlayed in ImageJ. No contrast enhancements or brightness levels were altered after acquisition.

Antibodies and reagents used for immunohistochemistry were as follows: anti-CtBP2/RIBEYE antibody (BD Transduction Laboratories #612044; 1:200 dilution); biotinylated-PNA (Vector Laboratories #B-1075; 1:500 dilution).

Images were taken directly superior and inferior of the optic nerve head and were gathered at 40× magnification. Three serial images were acquired on either side of the optic nerve leading to 6 total images gathered per eye. These images were then independently quantified by three individuals masked to treatment groups and the number of cones per image was averaged. The quantifications were averaged and divided by the image width to produce the reported average cones per 100 μm.

For immunohistochemistry on the whole flat-mount retinas, the retina was permeabilized using 0.3% Triton X-100 in PBS for 10 min at room temperature, blocked with blocking buffer containing 5% BSA, 5% normal goat serum, and 0.05% Triton X-100 in PBS at 4°C overnight. The following day, samples were washed in 1xPBS two times for 10 min in room temperature before incubation with secondary antibody streptavidin Alexa Fluor-568 conjugated and Hoechst 1:1000 (Thermo Fisher Hoechst 33342) at room temperature for 1 h tilted in a 2 mL Eppendorf tube. After washing in 1xPBS two times for 10 min at room temperature, samples were mounted with Fluoromount-G (Electron Microscopy Science #17984-25). Images were taken on a THUNDER Imager (Leica DM6B microscope equipped with a Leica DFC9000 GT camera), *z*-stacks were between 10–17 stacks at 1.5 μm and computational clearing of *z*-stacks was carried out using LAS X software. With the use of ImageJ, two rectangle boxes per retina were cropped to approximately 350 × 350 μm to obtain a uniform area in which to count cones on a flat mount. The image was converted to 8-bit and thresholded to isolate the cone staining on the image. Once these steps were completed, a particle analysis was conducted to count and label cones present in the cropped image. Cone density of buffer-injected or sham-punctured eye was normalized to its untreated fellow eye and reported as a percentage.

## 3 Results

### 3.1 Natural history of disease in the *Rs1*-KO mouse compared to WT BL/6 mouse

The features of the *Rs1*-KO mouse model were characterized. Cyst severity was quantified over time in the *Rs1*-KO mouse using a modified cyst severity scoring system, as previously described by Bush et al. (19). Figure 1 demonstrates typical OCT findings in the *Rs1*-KO mouse at p15, 1-, 2-, and 4-months of age, compared to a WT mouse (Figure 1A). Retinal cysts can be observed in the *Rs1*-KO mouse retina up to 3 months of age, in contrast to the organized, laminar retinal architecture of the WT mouse. Cysts are apparent as early as p15 with an average severity score of 2.75 out of 6 (Figure 1B). Cysts appear the most severe at 2- to 4-months old, hitting a local maximum at 3-months. Thereafter, cyst severity reduced with maturity.

At p15, *Rs1*-KO mice have comparable ONL thicknesses (65.56 μm) as mice that are WT (62.8 ± 6.23) (22), or heterozygous for the *Rs1* gene. In untreated *Rs1*-KO mice, significant thinning of the ONL occurred from p15 to p22 (Figure 1C, *p* < 0.0001), during which the ONL thickness was decreased by more than 37%. After 1-month, the ONL thickness in *Rs1*-KO mice did not significantly change, indicating that most of the photoreceptor cell loss occurred during eye maturation in this mouse model.

ERGs measure the electrical response of the retina *in vivo* to understand how the disease affects signal transmission in light and dark conditions. ERGs of *Rs1*-KO mice consistently demonstrated a reduction in function of rod-dependent and cone-dependent visual pathways compared to WT eyes. Figure 1 demonstrates typical ERG waveforms for this XLRS model at 2 and 6 months of age under light-adapted (5 Hz flicker) metrics (Figure 1D). In summary, features of the *Rs1*-KO mouse model resemble the clinical course in humans, including cyst formation, ONL thinning, and diminished electrical activity of retinal photoreceptors and bipolar cells observable on ERG. The phenotypes observed mirror those reported by Sieving et al. (14).

By administering gene therapy at 1-month of age, we aimed to determine if the intervention could mitigate the severity of cysts in the retina and subsequently improve the electrical signaling of retinal cells after the initial ONL thinning phase. Results from vector-treated and hypertonic buffer-injected eyes were compared to those of treatment naïve eyes (untreated).

### 3.2 Cyst severity is significantly reduced in vector-treated and hypertonic buffer-injected eyes at 1 MPI

**Figure 2:**
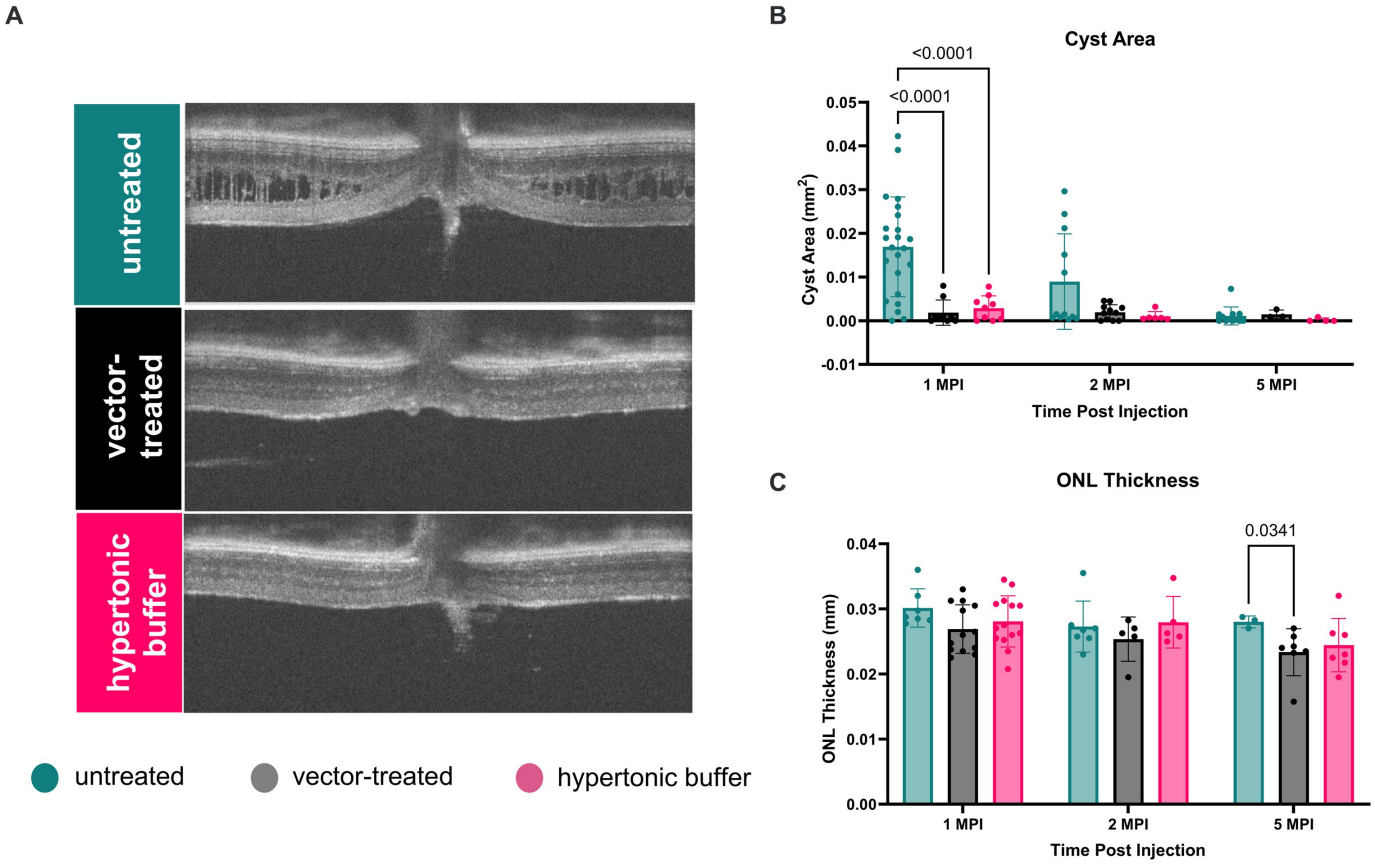
**(A–C)** Cyst severity is significantly reduced in vector-treated and hypertonic buffer-injected eyes at 1 month post injection (MPI). OCT images of vector-treated, hypertonic buffer-injected, and untreated eyes were collected at 1, 2, and 5 MPI. Volumetric scans were taken centering on the optic nerve. Representative OCT images demonstrate cyst changes at 1 MPI **(A)**. Cyst severity **(B)** was determined via manual segmentation of cyst area using the ImageJ software. Multiple comparisons were performed between every group at each time point. Non-significant comparisons were not annotated in the graphs. Vector-treated (*n* = 10) or hypertonic buffer-injected *Rs1*-KO eyes demonstrated significantly less cyst severity than untreated eyes **(B)** at 1 MPI. ONL thickness **(C)** remained stable from 1 to 5 MPI in all cohorts; however, at 5 MPI, vector treated eyes had statistically thinner ONLs than untreated eyes.

Using cyst area (mm^2^) of central OCT cross-sections as a measure of cyst severity, we observed that cysts were nearly completely ameliorated in vector-treated *Rs1*-KO eyes (*n* = 10) and hypertonic buffer-injected *Rs1*-KO eyes, in contrast to untreated eyes at 1 MPI (vector-treated: 0.001871 mm^2^, *p* < 0.0001; hypertonic buffer: 0.002878 mm^2^, *p* < 0.0001, untreated: 0.01691 mm^2^, Figures 2A,B). This reduction in cyst burden was particularly noteworthy because it coincided with the critical cyst developmental period around 2-months of age, a time when peak cyst formation is typically observed in *Rs1*-KO mice (Figure 1). This data suggests that the injection of either the vector or hypertonic buffer can effectively reduce cysts during a crucial phase of cyst development. Interestingly, there was no difference in the extent of cyst reduction between the vector and hypertonic buffer-injected eyes.

At subsequent time points, 2 and 5 MPI, since cyst severity naturally decrease in untreated *Rs1*-KO eyes (Figure 1B), we did not observe significant differences in cyst severity among untreated, vector-treated, or hypertonic buffer-injected eyes (14) There is no notable change in ONL thickness from 1 to 5 MPI in all cohorts; however, at 5 MPI, vector treated eyes had a statistically thinner ONL than untreated eyes (*p* = 0.0341) (Figure 2C).

Cysts spatially separate bipolar cells and photoreceptors, and it is hypothesized that the lower b-wave amplitudes and/or electronegative ERG phenotype associated with XLRS is a consequence of this spatial separation. To examine the potential influence of reduced cysts on retinal electrical signaling, we conducted ERGs under light-adapted and dark-adapted conditions.

### 3.3 Hypertonic buffer outperforms untreated and vector-treated eyes in light-adapted ERGs, but not in dark-adapted ERGs

ERGs were performed at 1, 2, 3, and 5 MPI to evaluate treatment efficacy, and ERG amplitudes in vector-treated *Rs1*-KO eyes (*n* = 10) or hypertonic buffer-injected *Rs1*-KO eyes (*n* = 15) were compared to those in their contralateral untreated *Rs1*-KO eyes.

#### 3.3.1 Light-adapted

**Figure 3:**
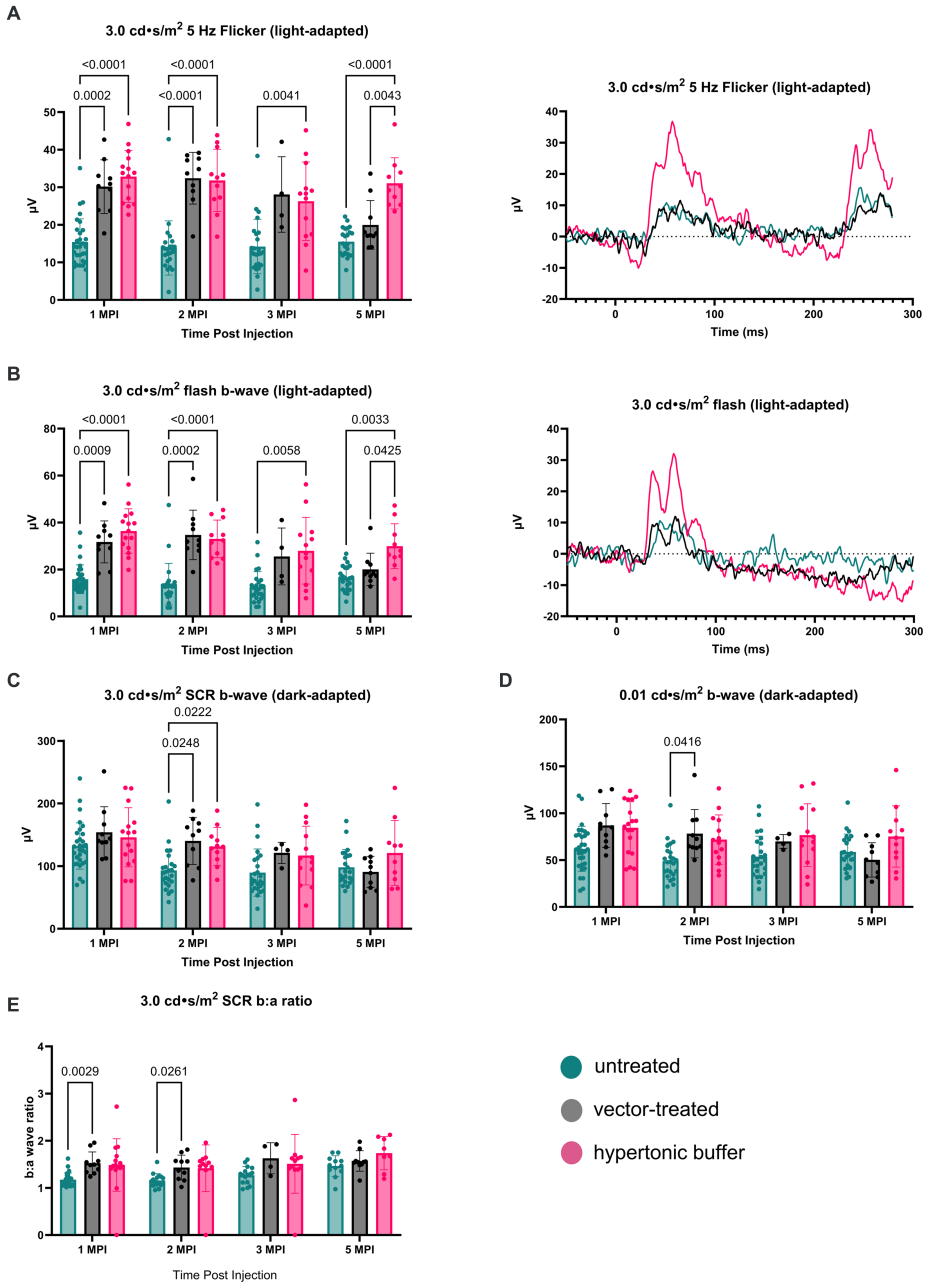
**(A–E)** Hypertonic buffer robustly outperforms untreated and vector-treated eyes in light-adapted ERGs. Cone-dependent retina function was measured using two separate light-adapted ERG assays—the 5 Hz flicker **(A)** and 3.0 flash **(B)**. ERG amplitudes are shown from 1-month post injection (MPI) to 5 MPI for vector-treated, hypertonic buffer-injected, and untreated eyes in *Rs1*-KO mice, and representative ERG traces at 5 MPI are illustrated. In response to both light-adapted assays, hypertonic buffer-injected eyes showed higher amplitudes than untreated and vector-treated eyes to 5 MPI **(A)**. The combined rod-cone function was measured by the 3.0 cd·s/m^2^ standard combined response (SCR) under dark-adapted conditions **(C)**. At 1, 3, and 5-MPI, there was no difference between vector-treated, hypertonic buffer-injected, or untreated eyes. Isolated rod function was measured using 0.01 cd·s/m^2^ dim flash after dark adaptation **(D)**. There was no persistent significant difference between hypertonic buffer-injected, isotonic buffer-injected, or untreated eyes in rod dominant ERG settings.

The retinal cone pathway was assessed using two light-adapted ERG assays: the 5 Hz flicker and the 3.0 flash. At 1 MPI, both vector-treated and hypertonic buffer-injected eyes showed significantly higher amplitudes, suggestive of superior cone function, compared to untreated eyes in the 5 Hz flicker assay (vector-treated: 30.17 ± 7.14 μV, *p* = 0.0002; hypertonic buffer: 32.86 ± 6.88 μV, *p* < 0.0001; untreated: 15.42 ± 6.17 μV, Figure 3A). This effect persisted until 2 MPI in vector-treated eyes (vector-treated: 32.42 ± 6.89 μV, *p* < 0.0001) after which amplitudes decreased and significance over untreated eyes was lost. Interestingly, hypertonic buffer injected eyes continued to have significantly higher amplitudes than untreated eyes to 5 MPI (hypertonic buffer: 31.05 ± 6.82 μV, *p* < 0.0001; Figure 3A).

Notably, at 5 MPI, hypertonic buffer-treated eyes demonstrated significantly higher amplitudes than vector-treated eyes (19.98 ± 6.49 μV, *p* = 0.0043), indicating a more robust and sustained rescue of cone function in the hypertonic buffer group compared to the low-dose AAV2/4-EF1α-*RS1* vector (Figure 3A). This data suggests that subretinal injection of the hypertonic buffer provides a more effective long-term improvement in cone function than the vector treatment.

Similarly, in the 3.0 flash assay, vector-treated eyes demonstrated significantly higher amplitudes, suggestive of superior cone function, compared to untreated eyes until 2 MPI (vector-treated: 34.70 ± 10.56 μV, *p* = 0.0002; hypertonic buffer: 33.04 ± 8.01 μV, *p* < 0.0001; untreated: 13.75 ± 8.85 μV, Figure 3B) after which time amplitudes began to decrease. However, hypertonic buffer-injected eyes exhibited a more robust and long-term improvement in function with higher amplitude ERGs persisting to 5 MPI (29.93 ± 9.57 μV, *p* = 0.0033), when compared to untreated eyes (16.43 ± 5.19 μV), and even demonstrated significantly higher amplitudes than vector-treated eyes at 5 MPI (20.03 ± 6.87 μV, *p* = 0.0425). This robust and sustained improvement in cone function observed in the hypertonic buffer group was an unexpected finding, indicating that the subretinal injection of the buffer alone enabled long-term benefits in retinal cone function. This phenomenon is specific to XLRS, as injection of a hypertonic buffer into eyes without XLRS typically reduces the ERG amplitudes due to surgical trauma (Supplementary Figure S1). Since injection of buffer alone causes an increase in b-wave amplitudes in the absence of restoring RS1 protein expression, this phenotype could be the consequence of cyst amelioration. Representative waveforms demonstrate these findings at 5 MPI (Figures 3A,B).

#### 3.3.2 Dark-adapted

To measure the electrical function of the eye in dark conditions, the 3.0 SCR and 0.01 dim flash ERG assays were utilized. At 1 MPI, no significant difference was observed in the b-wave amplitudes between untreated eyes and vector-treated or hypertonic buffer-injected eyes in the 3.0 SCR (vector-treated: 153.8 ± 40.87 μV, *p* = 0.3211; hypertonic buffer: 146.0 ± 47.17 μV, *p* = 0.5819, untreated: 132.0 ± 37.00 μV, Figure 3C), or 0.01 dim flash assays (vector-treated: 86.91 ± 23.39 μV, *p* = 0.0691, hypertonic buffer: 84.48 ± 27.73 μV, *p* = 0.0541, untreated: 62.01 ± 24.05 μV, Figure 3D). At 2 MPI, both the vector-treated and hypertonic buffer-injected eyes showed significantly higher amplitudes, suggestive of an improved combined function of rod and cone photoreceptors in the 3.0 SCR (vector-treated: 140.2 ± 37.56 μV, *p* = 0.0248, hypertonic buffer: 131.3 ± 30.43 μV, *p* = 0.0222, untreated: 93.07 ± 32.97 μV, Figure 3C). A similar effect was seen at 2 MPI in vector-treated eyes during the 0.01 dim flash assay (vector-treated: 78.12 ± 25.75 μV, *p* = 0.0416, Figure 3D). Under the 3.0 SCR b:a wave ratio, vector treated eyes had significantly higher b:a wave amplitude ratios than untreated eyes util 2 MPI (vector-treated: 1.43 ± 0.26 μV, *p* = 0.0261; untreated: 1.16 ± 0.14 μV, Figure 3E). However, these improvements were transient and were not maintained at 3 and 5 MPI. These results indicate that injection of low-dose AAV2/4-EF1α-*RS1* vector or hypertonic buffer did not have a notable long-term effect on rod-dependent retinal function, nor on rod-cone combined function as measured by the 3.0 SCR test. Improvements in ERG amplitudes after the injection of hypertonic buffer were most notable in cone-dependent ERGs.

Following the observed improvements in cone specific electrical pathways, we investigated whether these improvements were associated with differences in functional vision.

### 3.4 Vector-treated and hypertonic buffer-injected mice perform faster in a light-adapted swim assay than untreated *Rs1*-KO mice

**Figure 4:**
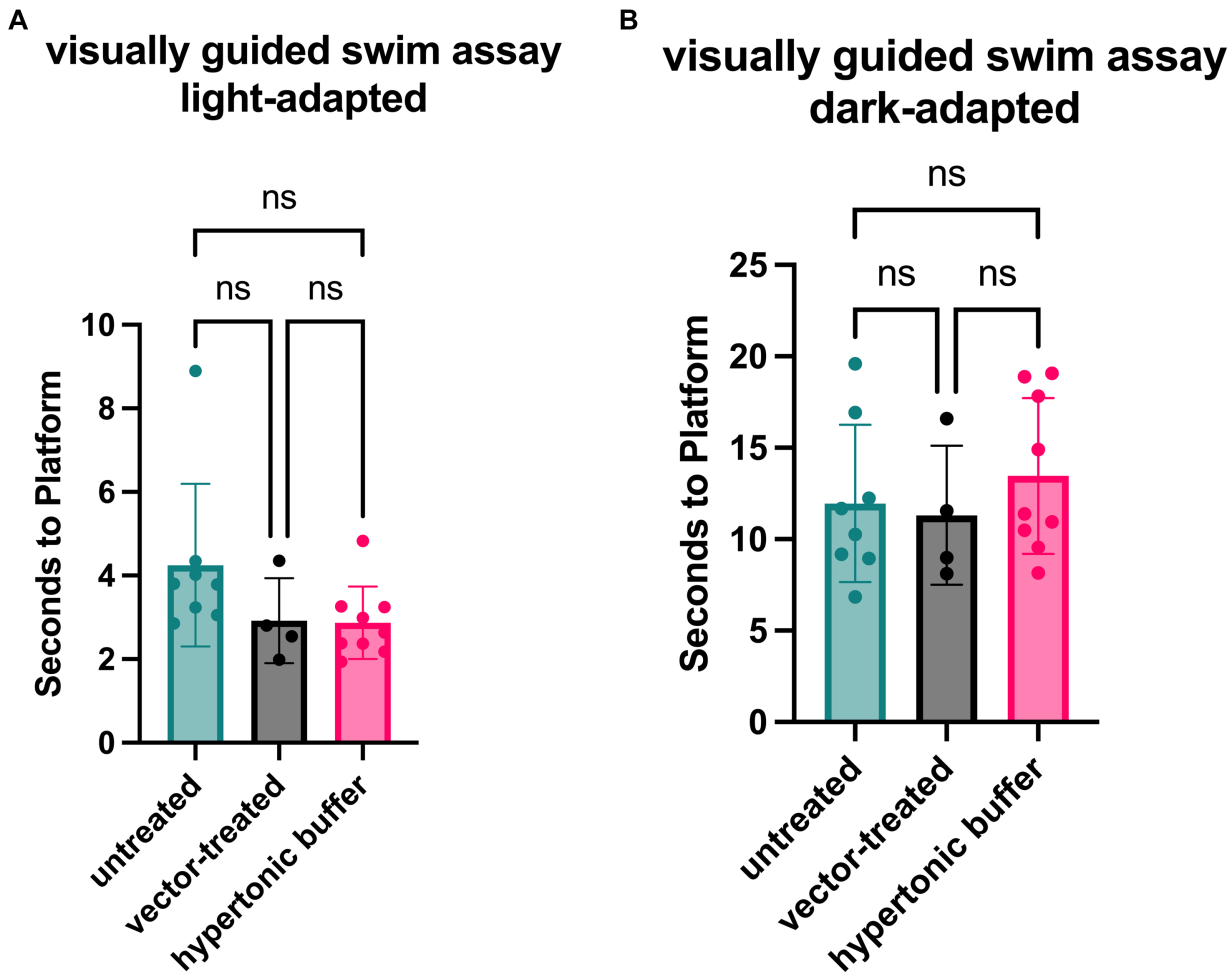
**(A,B)** Vector-treated and hypertonic buffer-injected *Rs1*-KO mice perform faster in a light-adapted swim maze than untreated *Rs1*-KO mice. Mice were placed in a swim maze and testing was performed under light-adapted **(A)** or dark-adapted **(B)** conditions. Time-to-platform was recorded. Each data point is representative of the average time-to-platform for a single mouse. In light-adapted conditions (untreated *n* = 8, vector-treated *n* = 4, hypertonic buffer *n* = 10.), we observed that vector-treated mice (2.92 s) and hypertonic buffer-injected mice (3.05 s) had a generally faster trend in average swim performance when finding a random platform than untreated mice (4.50 s) in lighted environments. In a dark-adapted environment, there is a less impactful effect on average time-to-platform between vector-treated (11.31 s), hypertonic buffer-injected (13.78 s), and untreated eyes (11.13 s), suggesting a limited effect on visual function in the dark **(B)**.

Functional vision in mice subretinally injected with the low-dose AAV2/4-EF1α-*RS1* vector or hypertonic buffer, as well as the untreated *Rs1*-KO mice, was measured using a VGSA. Mice between 5 to 7-months of age were placed in a pool and platform locations were randomly determined for each trial in light-and dark-adapted environments. One datapoint represents the averaged time it took for a mouse to reach the platform over 20 different trials using randomized platform positions. Administration of the vector or buffer was unilateral. In light-adapted conditions, we observed that vector-treated mice (2.92 s) and hypertonic buffer-injected mice (3.05 s) had a generally faster trend in average swim performance than untreated mice (4.50 s) in lighted environments (Figure 4A). These results confirm that the subretinal injection of vector or buffer alone has potential to benefit functional vision in the light, which correlates with the apparent reduction of cysts, and improved cone pathway ERG signaling. The trends in improvement of functional vision seen in vector-treated mice was comparable to that observed in buffer-injected mice in light-adapted testing conditions. There was less of an impact on functional vision in dark-adapted swim environments (Figure 4B).

Together, the long-term ERG data and functional vision assays suggest that subretinal injections of a hypertonic buffer solution alone led to significant reductions in cyst severity and cone-specific improvements in retinal function and functional vision. The improvements observed in buffer-treated eyes were comparable to, and in some instances superior to, those observed in vector-treated eyes, indicating that treatment with a low-dose AAV2/4-EF1α-*RS1* was not efficacious. The reason behind the functional rescue observed in *Rs1*-KO mice subretinally injected with buffer is not clear.

### 3.5 Investigation of the effect of tonicity on cone rescue

The injection of hypertonic buffer alone leads to significant improvements in cone-specific ERG amplitudes, light-adapted visual function, and retinal architecture in *Rs1*-KO mice. Surprisingly, the observed improvements in ERG amplitudes after buffer injection persisted for at least 5 MPI. Previous studies have demonstrated a temporary reduction in existing retinal cavities following the subretinal injection of a balanced salt solution; however, the long-term effects on visual function and electrical signaling of the retina has not been studied (23). Additionally, cystoid retinal conditions in XLRS patients can be modified by the application of diuretic ophthalmic solutions such as brinzolamide and acetazolamide but the connection between fluid reabsorption and cyst resolution is not well understood (8). Previous literature has suggested that the RS1 protein physically interacts with Na/K-ATPase and may act to mediate Na/K-ATPase activity, affecting signaling and ion gradients within the retina (24, 25).

The buffer solution, used both to store the AAV vector and used in the initial buffer injections, contained a high salt content and a greater than physiologic osmolarity. Therefore, we hypothesize that the tonicity of the injected buffer plays a role in promoting cyst resolution and improving retinal signaling. This hypothesis was investigated through a subretinal injection of an isotonic buffer. The isotonic buffer has a similar composition to the hypertonic buffer except possessing a lower concentration of NaCl. Additionally, to account for mechanical trauma to the eye during the injection procedure, a sham cohort was added and received a scleral puncture and introduction of a Hamilton needle subretinally, to mimic injection without the introduction of subretinal fluid, hereafter referred to as the “puncture” group. The endpoints of these two additional experimental groups also include ERG to measure retinal electrical function and OCT to measure retinal structure and cyst severity. The results for these tests are as follows.

### 3.6 Cyst area is reduced in hypertonic buffer and isotonic buffer-injected eyes, with more robust reduction observed in the hypertonic buffer group

To determine if the tonicity of the injection buffer ameliorated cysts post injection, OCT was used to image the retinal layers *in vivo*. Cyst severity was evaluated by the segmentation of total cyst area in the central OCT scan. Outer nuclear layer (ONL) thickness measurements were taken to assess photoreceptor cell survival.

**Figure 5:**
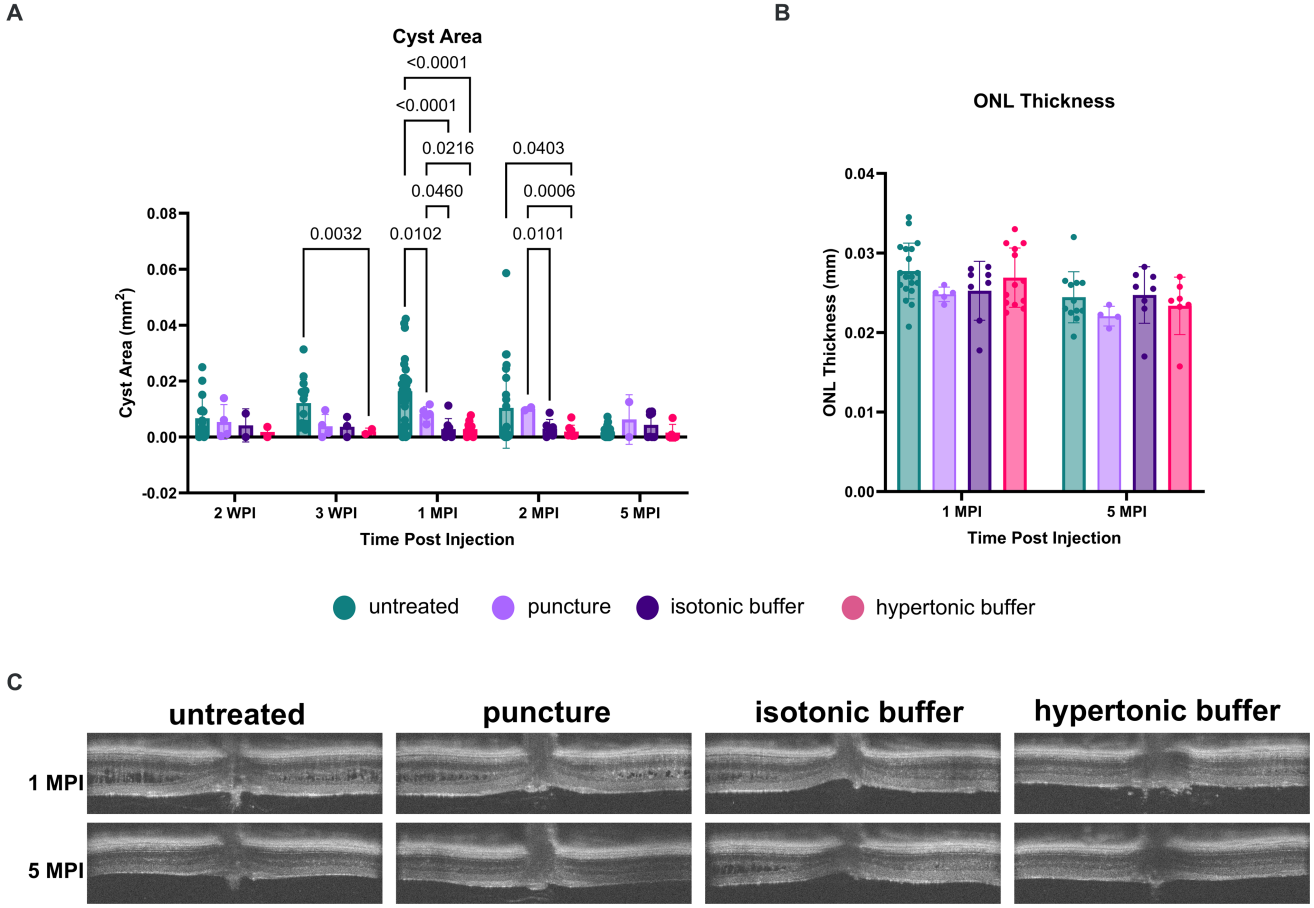
**(A–C)** Cyst area is reduced in hypertonic buffer and isotonic buffer-injected eyes, with greater reduction observed in the hypertonic buffer group. OCT images were collected at 2- and 3-weeks post injection (WPI), 1-month post-injection (MPI), and 2 MPI. Cyst area quantification **(A)** and outer nuclear layer (ONL) thickness **(B)** measurements were taken to assess cyst severity and photoreceptor cell survival, respectively. Representative OCT images demonstrate cysts at respective time points in all experimental groups **(C)**. Each data point is representative of one eye. At 3 WPI, there was less cyst severity in the hypertonic buffer-injected eyes compared to untreated eyes **(A)**. At 1 MPI, hypertonic and isotonic buffer-treated eyes both have less severe cysts than untreated and sham punctured eyes. This effect persisted until 2 MPI for the hypertonic buffer-injected eyes compared to untreated and sham punctured eyes. ONL thickness remains stable to 5 MPI and does not significantly differ between injection groups and untreated controls **(B)**.

While all groups receiving buffer injections as well as the puncture group had reduced cyst severity at 3 weeks post injection (WPI), the group receiving injections of the hypertonic buffer had the greatest reduction (Figure 5A). In the hypertonic buffer-treated group, cyst area was decreased by 83.77% compared to the cyst areas in untreated eyes (*p* = 0.0032). Cyst areas were reduced by 68.32 and 69.53% in the puncture group and the isotonic group, respectively (Figure 5A). While a sham puncture influenced cyst reduction, the greatest reduction was observed after the injection of the hypertonic buffer.

At 1 MPI, both hypertonic and isotonic buffer-treated eyes exhibited significantly less severe cysts than untreated or sham punctured eyes. In the hypertonic buffer-treated group, cyst area was decreased by 82.57% compared to the cyst area of untreated eyes (*p* < 0.0001), and 65.46% compared to cyst area of sham punctured eyes (*p* = 0.0216, Figure 5A). This implies that subretinal injection of the buffer solution caused cyst reduction, and the reduction is not solely caused by the surgical puncture. In the isotonic buffer-treated group, cyst area was decreased by 82.53% compared to the cyst area of untreated eyes (*p* < 0.0001, Figure 5A), and 65.38% compared to cyst area of sham punctured eyes (*p* = 0.0460, Figure 5A). At 1 MPI, sham punctured eyes showed a 49.54% reduction in cyst area compared to untreated eyes (*p* = 0.0102).

At 2 MPI, there is no longer a significant difference between cyst severity in the puncture group versus in untreated eyes, indicating that the alleviation of cysts in the sham puncture group was transient. In contrast, the reduction in cyst severity persisted to 2 MPI in hypertonic buffer-injected eyes compared to untreated eyes (81.33% reduction, *p* = 0.0403) and eyes that received only the sham surgical puncture (80.80% reduction, *p* = 0.0006). At 2 MPI, isotonic buffer-treated eyes also demonstrated a significant reduction in cyst severity compared to sham punctured eyes (70.03% reduction, *p* = 0.0101). Isotonic buffer-injected eyes did not have significantly reduced cyst severity compared to untreated eyes at 2 MPI (*p* = 0.1145). Representative OCT images of all experimental groups at 1 and 5 MPI are demonstrated in Figure 5C.

In summary, hypertonic buffer-injected eyes demonstrated an early and sustained reduction in cyst severity, starting as early as 3 WPI and persisting to 2 MPI. Additionally, the reduction in cyst severity was slightly greater in hypertonic buffer-injected eyes than in isotonic buffer-injected eyes at 3 WPI and 1 MPI.

Though cysts were decreased at early timepoints, ONL thickness was not different across time points between hypertonic buffer-injected, isotonic buffer-injected, sham punctured, and untreated eyes, suggesting that there is no difference in photoreceptor survival using this method.

### 3.7 Hypertonic buffer-injected eyes have a more robust cone rescue on light-adapted ERG over untreated, sham punctured, and isotonic buffer-injected eyes

#### 3.7.1 Light-adapted

Following an observed reduction in retinal cysts after subretinal injection of a hypertonic buffer, we investigated whether the improvement in cysts paralleled a similar improvement in electrical function of the retina.

Cone function was measured using two separate light-adapted ERG assays; the light adapted (LA) 3.0 flash and 5 Hz flicker.

**Figure 6:**
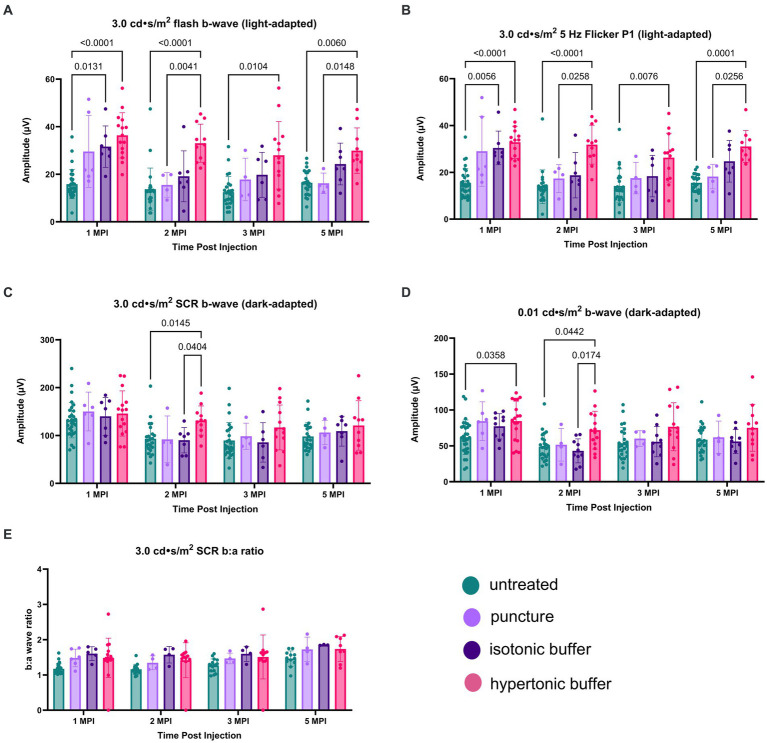
**(A–E)** Hypertonic buffer-injected eyes experience a robust rescue of cone functioning. Cone-dependent retina function was measured using two separate light-adapted ERG assays—the 3.0 cd·s/m^2^ flash **(A)**, and the 5 Hz flicker **(B)** in hypertonic buffer, isotonic buffer, and sham punctured eyes in *Rs1*-KO mice. Each data point represents one eye. ERG amplitudes are shown from 1-month post injection (MPI) to 5 MPI. In both light-adapted assays **(A,B)**, hypertonic and isotonic buffer-injected eyes showed higher amplitudes compared to untreated eyes at 1 MPI. Hypertonic buffer-injected eyes showed improvements over untreated and sham punctured eyes until 5 MPI. The combined rod-cone function was measured by the 3.0 cd·s/m^2^ SCR under dark-adapted conditions **(C)**. Rod-dependent retina function was measured by the 0.01 dim flash **(D)**. In the 0.01 dim flash, hypertonic buffer-injected eyes showed improvements over untreated at 1 MPI and over both untreated and isotonic buffer-injected eyes at 2 MPI. However, significant differences over time were not observed.

Under the light-adapted 3.0 flash assay, both hypertonic buffer and isotonic buffer-injected eyes showed significantly higher amplitudes, indicative of better cone cell functioning, compared to untreated eyes at 1 MPI (hypertonic buffer: 36.37 ± 9.50 μV, *p* < 0.0001; isotonic buffer: 31.62 ± 8.71 μV, *p* = 0.0131; untreated: 15.84 ± 6.14 μV, Figure 6A). At this time point, eyes that received the sham puncture also had increased amplitudes (29.59 ± 15.10 μV). Hypertonic buffer-injected eyes continued to demonstrate improved cone functioning over untreated eyes throughout the study period, including at the study endpoint at 5 MPI (hypertonic: 29.93 ± 9.57 μV, *p* = 0.0060; untreated: 16.43 ± 5.19 μV, Figure 6A). At the endpoint, eyes that received puncture did not have a significant difference compared to untreated eyes, indicating that the effect of a surgical puncture on RS1 phenotypes was transient. Additionally, hypertonic buffer-injected eyes showed significant improvements over sham punctured eyes at 2 MPI (hypertonic: 33.04 ± 8.01 μV, *p* = 0.0041; sham punctured: 15.51 ± 5.17 μV) and 5 MPI (hypertonic: 29.93 ± 9.57 μV, *p* = 0.0148; sham punctured: 16.21 ± 4.29 μV, Figure 6A).

Similar findings were observed in the second, cone-dependent assay, the 5 Hz flicker. Both hypertonic buffer and isotonic buffer-injected eyes showed significantly higher amplitudes compared to untreated eyes at 1 MPI (hypertonic buffer: 32.86 ± 6.88 μV, *p* < 0.0001; isotonic buffer: 30.43 ± 7.19 μV, *p* = 0.0056; untreated: 15.42 ± 6.17 μV, Figure 6B). However, the effect of isotonic buffer injection was not as notable as that observed in hypertonic buffer injected eyes, indicating the tonicity of the buffer could play a role in this observation. In contrast, hypertonic buffer-injected eyes continued to have significantly higher amplitudes, indicating sustained improved cone function, compared to untreated eyes until the study endpoint at 5 MPI (hypertonic buffer: 31.05 ± 6.82 μV, *p* < 0.0001; untreated: 15.52 ± 3.71 μV, Figure 6B). Additionally, hypertonic buffer-injected eyes had significantly higher amplitudes than sham punctured eyes at 2 MPI (hypertonic: 31.82 ± 8.29 μV, *p* = 0.0258; sham punctured: 17.36 ± 5.93 μV, Figure 6B) and 5 MPI (hypertonic: 31.05 ± 6.82 μV, *p* = 0.0256; sham punctured: 18.20 ± 5.24 μV, Figure 6B).

These results show that injection of a hypertonic buffer significantly improved the amplitudes of cone-dependent ERGs, and this effect persists even when cysts are naturally resolved due to age in the *Rs1*-KO mouse model. The injection of an isotonic buffer also had a beneficial effect, although the magnitudes of improvement were not comparable to those in hypertonic buffer-injected eyes. A transient effect was observed in eyes that received the scleral puncture only (without the injection of a buffer), indicating that the effect of surgical manipulation on cyst and retinal function was transient.

Together, these results show that the introduction of a subretinal buffer solution has unexpected beneficial effects on cyst severity and cone retinal function in eyes affected by XLRS, and the observed effect is long-term. Additionally, the tonicity of the injected buffer solution may play a role in the observed phenomenon.

#### 3.7.2 Dark-adapted ERG

Combined rod-cone function was measured by the 3.0 SCR, and isolated rod function was measured using the 0.01 dim flash. In the 3.0 SCR, hypertonic buffer-injected eyes showed improved amplitudes over untreated (*p* = 0.0145) and isotonic buffer-injected eyes (*p* = 0.0404) at 2 MPI (Figure 6C). In the 0.01 dim flash, hypertonic buffer-injected eyes showed improvements over untreated at 1 MPI (*p* = 0.0358, Figure 6D) and over both untreated (*p* = 0.0442) and isotonic buffer-injected (*p* = 0.0174) eyes at 2 MPI (Figure 6D). Hypertonic buffer-injected eyes did not show improvements over sham punctured eyes in dark-adapted assays. There was no difference between experimental groups in the SCR b:a wave ratio (Figure 6E). These findings suggest that hypertonic buffer-injection may lead to short term rescue of rods and that this effect may be influenced by the tonicity of the buffer.

### 3.8 Peak cyst severity on OCT is negatively associated with cone function on ERG at 5 MPI

Hypertonic buffer-injected eyes had both the lowest cyst severity and the greatest ERG improvements in cone-dominant electrical pathways. To explore this finding, we investigated whether cyst severity is truly associated with a long-term impairment in electrical signaling of the retina. The correlation between peak cyst severity (the highest cyst area reached by the eye across different timepoints) and long-term retinal function (ERG amplitudes at 5 MPI) were analyzed, and included hypertonic buffer-injected, isotonic buffer-injected, sham punctured and untreated eyes. Generally, as part of the natural disease course in this XLRS mouse model, retinal cysts hit a local maximum of severity between 2- and 4-months of age (Figure 1B). The presence of a negative correlation between peak cyst severity and 5 MPI ERG amplitudes would imply that the degree of cyst severity during critical times during disease course correlated with long-term electrical function in the retina. By looking at long-term outcomes, we will determine if the reduction of cysts could influence long-term retinal function beyond any short-term benefits due to bringing the retinal layers closer together. Additionally, at 5 MPI there are little to no cysts present in untreated mice based on natural history and could not explain the variation in ERG amplitudes at that time.

**Figure 7:**
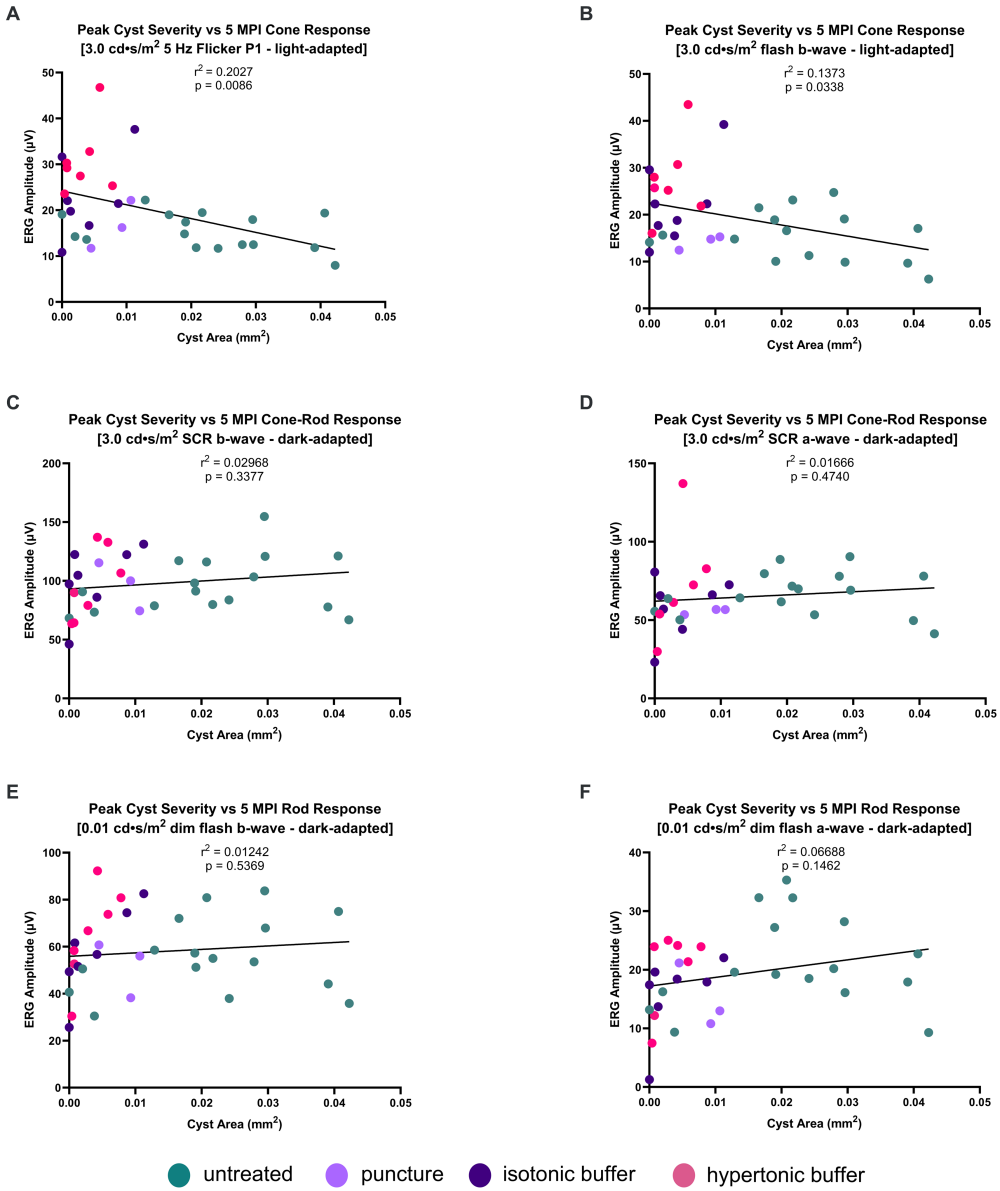
**(A–F)** Peak cyst severity on OCT is negatively associated with cone function on ERG at 5 MPI. Peak cyst severity of hypertonic buffer-injected, isotonic buffer-injected, sham-punctured and untreated eyes was determined by comparing the cyst area measurements of each eye at 1, 2, and 5 MPI. The cyst area of each eye representing their peak severity was correlated with its ERG amplitudes at 5 MPI. Each dot represents one eye. A negative correlation was found between cyst area and ERG amplitudes in the light-adapted 5 Hz flicker and 3.0 cd·s/m^2^ flash **(A,B)**. Little to no correlation was found between peak cyst area and ERG amplitudes in dark-adapted 0.01 cd·s/m^2^ or 3.0 cd·s/m^2^ SCR **(C–F)**. These findings suggest increasing cyst area has greater impact on impairing cone electrical function, than rod photoreceptors.

In light-adapted, cone-dominant, ERG metrics we found ERG amplitudes are negatively correlated with peak cyst severity in both the 5 Hz flicker and 3.0 flash (5 Hz flicker: *r*^2^ = 0.2027, *p* = 0.0086; Figure 7A) (3.0 flash b-wave, *r*^2^ = 0.1373, *p* = 0.0338; Figure 7B). These findings suggest that having a greater cyst area at any time during the disease course of a mouse is strongly associated with impaired signaling in cone-dominant electrical pathways long-term.

On dark-adapted, rod-dominant ERG metrics, there is no correlation between peak cyst area in either the a-wave or b-wave metrics (3.0 SCR b-wave: *r*^2^ = 0.02968, *p* = 0.3377; Figure 7C) (3.0 SCR a-wave: *r*^2^ = 0.01666, *p* = 0.4740; Figure 7D) (0.01 dim flash b-wave: *r*^2^ = 0.01242, *p* = 0.5369; Figure 7E) (0.01 dim flash a-wave: *r*^2^ = 0.06688, *p* = 0.1462; Figure 7F). Cyst severity, or the reduction thereof, does not appear to be associated with long-term rod signaling.

The negative correlation observed between cone-dominant ERG amplitudes and cyst severity indicates that having had severe cysts at any point in time is correlated with possessing lower cone-dependent ERG amplitudes over time. However, this correlation analysis cannot distinguish whether this is due to a decreased number of cones or possessing lower cone function. To determine whether there is a difference in the number of cone photoreceptor cells, we used an immunofluorescence assay to quantify cone density in eyes injected with hypertonic buffer compared to untreated eyes.

### 3.9 Increased cone density at 6 to 7-months old suggests cone preservation following injection of buffer solution

ONL thickness, a typical measure of photoreceptor cell survival, was not significantly different between eyes that received the sham surgical puncture, buffers with different tonicity, and untreated eyes (Figure 5B). In the setting of a low overall percentage of cones in the mouse retina (2.8% of total photoreceptors) (26), the impact of the injections on cone survival may not be accurately determined by measuring ONL thickness on conventional OCTs.

**Figure 8:**
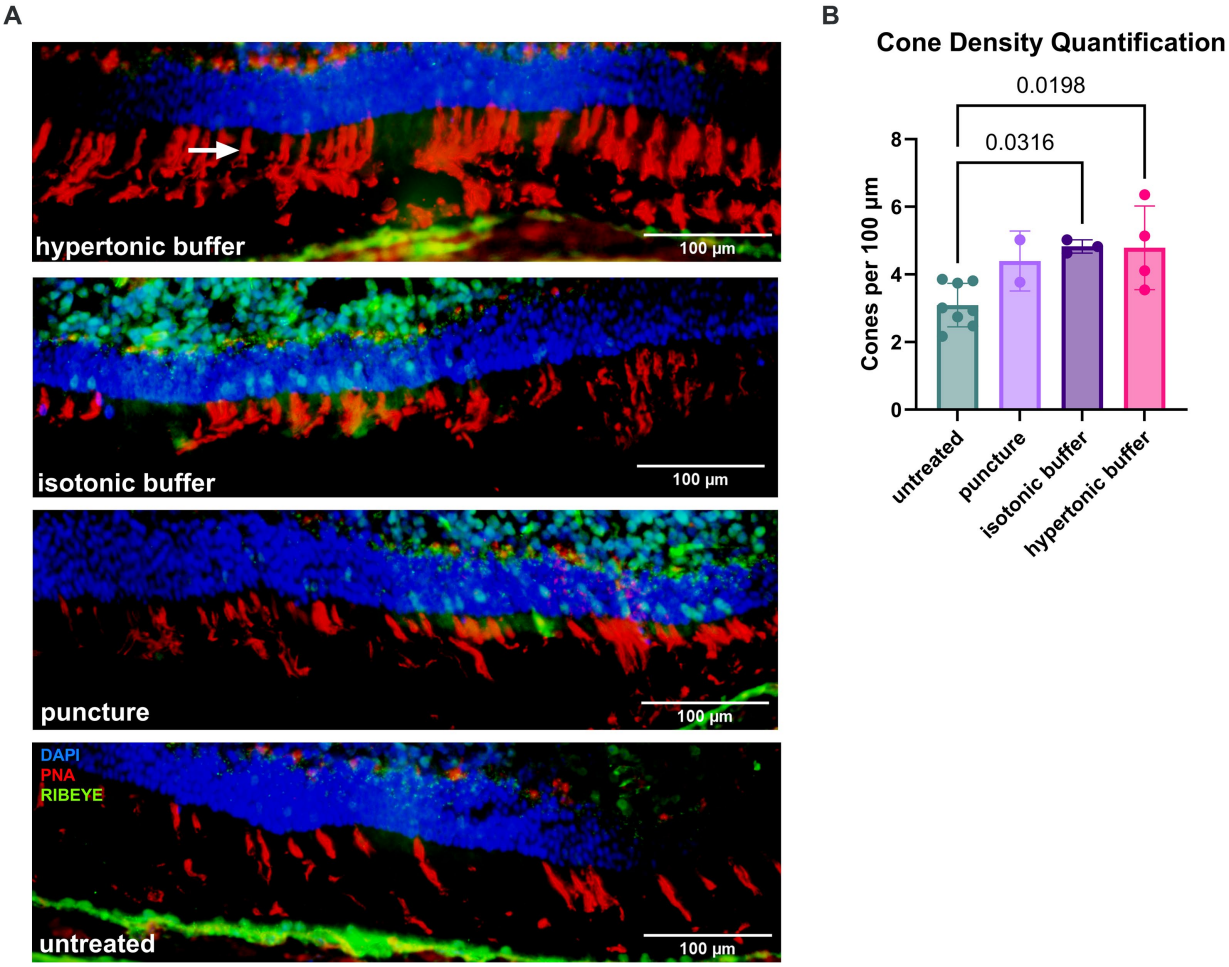
**(A,B)** Increased cone density at 5 months after subretinal injections of a hypertonic or an isotonic buffer. Retinal sections were collected from untreated *Rs1*-KO eyes, hypertonic buffer-injected eyes, isotonic buffer-injected eyes, and sham puncture eyes at approximately 6-months of age. Sections were stained by PNA to visualize cone outer segments. The number of cone outer segments per 100 μm of the retina was quantified by three participants masked to treatment groups, and quantifications by these three participants were averaged. Representative immunofluorescent staining (red: PNA) of cone outer segments (arrow), demonstrating increased density of cones in hypertonic buffer-injected and isotonic buffer-injected eyes compared to untreated eyes with sparse cone staining **(A)**. Synapses were visualized by an anti-RIBEYE antibody (green). Each data point represents the averaged cone density of one eye. Hypertonic buffer-injected eyes and isotonic buffer-injected eyes show significantly higher cone density per 100 μm than untreated *Rs1*-KO eyes **(B)**.

Immunohistochemistry was performed to measure cone cell survival. Retinal cryosections were stained with biotinylated-PNA, a marker for cone outer segments and pedicles (Figure 8A). The number of PNA-positive cone outer segments were quantified for 6 images per eye and the number of cones per 100 μm of the retina was computed. Untreated *Rs1*-KO eyes had a mean of 3.09 cones per 100 μm, hypertonic buffer-injected eyes had a mean of 4.79 cones per 100 μm, isotonic buffer-injected eyes had a mean of 4.83 cones per 100 μm, and sham punctured eyes had a mean of 4.39 cones per 100 μm. Both hypertonic buffer-injected, and isotonic buffer-injected eyes had significantly higher cone density than untreated *Rs1*-KO eyes, suggesting improved cone survival compared to untreated eyes (hypertonic buffer vs. untreated: *p* = 0.0198; isotonic buffer vs. untreated: *p* = 0.0316; Figures 8A,B). While both buffer-injected eyes had significantly more cones than untreated eyes, only hypertonic buffer-injected eyes showed significant improvements in light adapted ERG metrics. This suggests some mechanism beyond cone preservation could be responsible for the substantial improvements in ERG metrics seen in hypertonic buffer-injected eyes. Cone counts in specific regions of the retina inferior or superior to the optic nerve can be appreciated in Supplementary Figure S2.

At 9 to 10 MPI, flat mounts of buffer injected, sham punctured, and untreated eyes were collected to determine if cone preservation persisted to later time points. The number of cones in two separate quadrants of an area approximately 350 μm^2^, were quantified using ImageJ software, and the averaged cone density for that eye is reported. Cone density in the injected or sham punctured eye was normalized to the cone density in the untreated fellow eye and reported as a percent. Results of this experiment show a similar trend as manual cone counts derived at 6–7 months old, demonstrating that subretinal injections of buffer not only attenuate cysts in the short term but also lead to greater cone survival over time in the *Rs1*-KO mouse model (Supplementary Figure S3).

Together, this data shows that the subretinal injection of a buffer solution partially ameliorated intra-retinal cysts and leads to improved amplitudes in cone-specific ERGs as well as in functional light-adapted vision. The beneficial effect of the buffer solution after subretinal administration was partially mediated by the osmolarity of the buffer solution, with hypertonic buffer-injected eyes showing a more pronounced effect across all metrics.

## 4 Discussion

Human trials of XLRS gene therapy are presently underway, working to overcome challenges associated with ocular immune reactions following intravitreal injection in past studies (11, 12). Our study initially aimed to explore the potential of a low-titer gene therapy in circumventing such immune reactions.

Initially, a subretinal injection of a 2 × 10^9^ vg/mL dose of AAV2/4-EF1α-*RS1* in *Rs1*-KO mice did improve functional metrics such as cyst severity and cone electrical functioning, when compared to untreated *Rs1*-KO controls. However, it was surprising to observe that eyes injected with a hypertonic buffer as a sham injection also demonstrated improvements across all metrics compared to untreated *Rs1*-KO eyes. Even more unexpectedly, these eyes outperformed low-dose vector-treated eyes in cone electrical signaling and cyst severity scoring metrics, persisting to 5 MPI.

Previous studies utilizing higher titers delivered subretinally and intravitreally with an AAV2/4 vector and CMV promoter (27), and with a tYF modified capsid AAV (28) have demonstrated robust rescue of the retinal phenotype suggesting that sufficient RS1 expression is necessary to achieve optimal efficacy. However, toxicity was observed in some mice when the optimal dose was exceeded (28), highlighting the importance of understanding dose-response relationships in gene therapy for treating RS1. Considering the similarities in performance between the vector-treated and hypertonic buffer-injected eyes in the current study, it can be inferred that the efficacy observed with this very low dose vector was primarily attributed to its diluent buffer. Dose escalation in gene therapy is often accompanied by undesirable immunogenicity, yet a reduction of dose often results in loss of efficacy or sub-therapeutic outcomes. Together, these studies elucidate the tradeoff between achieving therapeutic efficacy and toxicity risks. However, the unexpectedly robust effect of the hypertonic buffer alone was not anticipated. While reductions in cysts for short periods following subretinal injection of a balanced salt solution have been reported in this model previously, this phenomenon has not been thoroughly explored (23).

To elucidate the mechanism of buffer-induced phenotypic rescue, ERG and OCT experiments were repeated after the subretinal injection of an isotonic buffer instead of the hypertonic buffer. Additionally, a sham group was added involving a scleral puncture to mimic injection without the introduction of subretinal fluid. The creation of a puncture in the sclera during the subretinal injection procedure causes leakage of a small amount of intra-ocular fluid, thus potentially lowering the intraocular pressure. Therefore, the addition of the sham puncture group accounts for this surgical effect.

Interestingly, hypertonic buffer-injected eyes exhibited a reduced cyst burden at 3 WPI, compared to untreated *Rs1*-KO eyes. By 1 MPI, a critical time point in cyst development as evidenced by natural history data (Figure 1B), eyes across all experimental groups—hypertonic buffer, isotonic buffer, and sham puncture, —had a reduction in cyst severity compared to untreated *Rs1*-KO eyes. However, only hypertonic buffer-injected eyes maintained a reduction in cyst severity over untreated *Rs1*-KO eyes to 2 MPI. At 2 MPI, isotonic and hypertonic buffer-injected eyes had a more significantly reduced cyst burden than the sham puncture group, suggesting the introduction of either buffer solution has a greater effect on reducing cyst burden, with a slightly greater effect observed in the hypertonic buffer-injected eyes. An overall decrease in cysts was evident at 5 MPI across all groups, consistent with disease progression.

On ERG, isotonic buffer and hypertonic buffer-injected eyes showed improvement in cone-specific electrical signaling. However, the improvements were more pronounced in eyes that received the hypertonic buffer, persisting till 5 MPI. The combined effect of ERG and OCT experiments suggests the tonicity of the injected solution appears to play a minor yet important role in reducing overall cyst severity and enhancing the function of retinal cells, specifically cones, post-injection. The phenotypic improvements observed after the eyes received a sham puncture were more transient in nature compared to buffer-injected eyes.

Paradoxically, the improvements in cone-specific ERGs persisted despite age-related cyst resolution, and hypertonic buffer-injected eyes continued to possess higher ERG amplitudes to the study endpoint at 5 MPI. Age-related cyst resolution in this model accompanies a progressive loss of the laminar structure as seen on OCT. The thicknesses of the ONLs were not different between the experimental groups of mice, so our understanding of these functional improvements seen in hypertonic buffer-injected eyes long after cysts have naturally resolved remains unclear. Since cones are reported to represent only 2.8 percent of photoreceptors in mouse retinas, the effect of the injection on cone cell survival would not be reflected by ONL thickness measurements (26). The low cone-to-rod ratio and absence of a cone-rich region in the mouse retina present a limitation to this study, making it challenging to use mouse models for direct comparisons to human retinal conditions.

Immunohistochemistry revealed significantly higher cone density in both hypertonic buffer-injected and isotonic buffer-injected eyes compared to untreated eyes at 6 to 7-months, which could explain the improvement in cone specific ERG amplitudes and improvements in functional vision in light adapted environments. This finding suggests a potential cone-preserving mechanism is associated with the subretinal introduction of buffer, most impressively from the subretinal injection of a hypertonic buffer. While this does not fully explain the results as there is not a significant difference between hypertonic-and isotonic buffer-injected eyes, it does suggest that cone preservation at least partially underlies the observed improvements. The additional improvements seen in hypertonic buffer-injected eyes could be due to improved synaptic function, but further experiments are necessary to elucidate the mechanism further. Eyes were also collected and stained at 9 to 10-months for cone quantification. While hypertonic and isotonic buffer-injected eyes continued to show a trend of higher cone counts compared to untreated eyes, the difference was no longer statistically significant (Supplementary Figure S3).

The long-term beneficial effects on XLRS phenotypes, including cone signaling, functional vision, and cyst severity, after the subretinal injection of a hypertonic buffer has not been previously reported. Previous studies propose that *RS1* might have additional functions outside of its structural function and play a role in governing fluid distribution across retinal layers, potentially by engaging with Na/K-ATPase channels via a binding mechanism (24, 25). In the absence of a functional protein, this pathway has the potential to become dysregulated, resulting in the formation of fluid filled retinal cysts and creating a spatial separation of bipolar and photoreceptor cells. Despite the incomplete understanding of the mechanism, this phenomenon could provide an explanation for the observed benefits of carbonic anhydrase inhibitors (CAIs) in mitigating cyst development and enhancing visual function among XLRS patients (2).

Systemic infusions of hyperosmolar fluid have been shown to reduce edema in other organs, like the brain, in the setting of acute cerebral injury (29). With this precedent, strategies leveraging fluid exchange to promote the resolution of cysts in edematous retinal conditions merit further exploration. Aquaporins (AQP) represent a family of water-transporting channels with a pivotal role in upholding water homeostasis across various organ systems. Previously identified in the inner nuclear layer of the retina, studies suggest they are involved in regulating retinal water balance (30). When AQP was knocked out in mouse models, electroretinograms (ERGs) demonstrated decreased b-wave amplitudes and delayed latency in a series of ERGs with increasing light intensity (30). These findings are parallel to our observations in the *Rs1*-KO mouse natural history and underscore the connection between the electrical function of the eye and the fluid homeostasis of the retina.

Currently, these data suggest that the injection of a buffer that is hypertonic to the physiological environment best alleviates intraretinal cysts, promotes the survival of cone photoreceptors, and preserves retinal function in a mouse model of XLRS. Furthermore, these data suggested that managing the severity of cysts can have a long-term impact on the health of the retina. Analyzing the relationship between the peak severity of cysts in each animal and their long-term functional outcomes on ERG revealed a negative correlation between cyst severity and light-adapted ERG metrics. This correlation was seen regardless of the treatment cohort (untreated, hypertonic buffer, isotonic buffer, or sham puncture), suggesting that the light-adapted metrics, and thus cone function and/or survival, are closely related to severity of cysts. In the setting of greater cyst severity, cone function is more attenuated than rod function. This also suggests that long term cone function can be predicted by the degree of cyst reduction achieved by treatment, specifically at early time points when cysts are expected to be most severe. These results suggest the successful management of cysts can impact the long-term health and function of the retina.

While we have not determined the exact cause of the more robust increase in cones than rods observed in our study, several studies have shown that differing physiological states, such as hypoglycemia or hypoxia, affect rods and cones differently (31). Additionally, studies have shown that under certain conditions, infiltrating microglia selectively target rod photoreceptors for phagocytosis (32). Cones may be more sensitive to synaptic disruptions due to schisis. In our study, it remains unclear whether the degree of cyst resolution, the timing of cyst resolution, or another unmeasured factor contributes to the observed increases. The known differing sensitivities of rod and cone photoreceptors suggests that the factors leading to cell death in these photoreceptors may differ and could explain the cone-specific improvements observed in our results.

The relationship between cyst reduction and enhanced cone survival has not been previously reported and could have significant clinical implications. Managing XLRS symptoms using topical agents such as brinzolamide has been found to reduce cysts and have beneficial effect on visual function (8). However, whether managing cysts in XLRS could have long-term benefits on cone specific function is not clear. Our study provides compelling evidence that the attenuation of cysts may promote the survival of cones in the retina, and lead to better electrical cone signaling and functional vision in the long-term.

This study has provided evidence that the management of cysts through an osmolarity-related mechanism could have beneficial effects and could guide control arms of future gene therapy interventions. Hyperosmolar solutions could potentially have a role in treatment of XLRS, either as diluent for gene therapy vectors or on their own. This observation underscores the necessity of including a control group receiving the diluent buffer when evaluating the efficacy of a gene therapy vector in JXLR, as the buffer has unexpected effects on RS1 phenotypes. The effect of the buffer is specific to the XLRS murine phenotype and not a generic effect seen in all mouse strains. Improvements in cone function and survival, cyst severity, and light-adapted visual function after hypertonic buffer-injection is a surprising finding and suggests tonicity is a mediator of this effect. It can be speculated that this effect is due to the shifting of fluid out of the retinal space, where cysts form, and into the subretinal space where the choroidal vasculature can reabsorb cystic fluid. Although the exact mechanism is unknown, this study also demonstrates that lower peak cyst severity at critical cyst development is associated with improved electrical function, improvements in light-adapted visual acuity, and greater preservation of cones. This effect is partially modulated by the tonicity of the buffer and is most prominent in eyes injected with a hypertonic buffer. These findings have the potential to open new doors in the development of therapies for patients with XLRS and could shine a light on how fluid balance influences cyst resolution and disease severity.

## Data availability statement

The original contributions presented in the study are included in the article/Supplementary material, further inquiries can be directed to the corresponding author.

## Ethics statement

The animal study was approved by the National Institute of Health in the Guide for the Care and Use of Laboratory Animals and Institutional Animal Care and Use Committee. The study was conducted in accordance with the local legislation and institutional requirements.

## Author contributions

EG: Conceptualization, Data curation, Formal analysis, Investigation, Methodology, Project administration, Supervision, Validation, Writing – original draft, Writing – review & editing. JT: Conceptualization, Data curation, Formal analysis, Investigation, Methodology, Project administration, Supervision, Validation, Writing – original draft, Writing – review & editing. EK: Conceptualization, Data curation, Formal analysis, Investigation, Writing – review & editing. SS: Data curation, Formal analysis, Investigation, Methodology, Writing – review & editing. JL: Writing – review & editing, Data curation, Investigation, Methodology, Writing – original draft. SB: Writing – review & editing, Conceptualization, Visualization. BL: Investigation, Writing – review & editing, Data curation. SM: Writing – review & editing, Investigation, Data curation. AM: Writing – review & editing, Investigation. SH: Writing – review & editing, Data curation. YH: Conceptualization, Project administration, Resources, Supervision, Validation, Visualization, Writing – review & editing, Data curation, Formal analysis, Investigation, Methodology. AD: Conceptualization, Funding acquisition, Project administration, Resources, Supervision, Validation, Visualization, Writing – review & editing.

## Supplementary material

SUPPLEMENTARY FIGURE S1 **(A–C)** Subretinal injection of hypertonic buffer alone does not enhance ERG amplitudes in mice without XLRS. To determine if the introduction of a hypertonic buffer has a stand-alone effect on ERG amplitudes, a cohort of mice without XLRS were subretinally injected with the buffer. ERGs were collected at 2, 3, and 6 MPI. Cone function was assessed using two light-adapted ERG assays: the 5 Hz flicker and the 3.0 cd·s/m² flash. In response to both the 3.0 cd·s/m² flash **(A)** and 5 Hz flicker **(B)** assays, hypertonic buffer-injected eyes showed no significant change in amplitude compared to untreated eyes. This demonstrates that the hypertonic buffer alone does not enhance ERG metrics, confirming that the observed improvements in cone-specific functioning in hypertonic buffer-injected *Rs1*-KO eyes are genuine. Representative waveforms at 6 MPI illustrate these findings, even indicating a pattern of mild reduction in amplitude associated with the subretinal injection of hypertonic buffer **(C)**.

SUPPLEMENTARY FIGURE S2 **(A–C)** Spatial distribution of cone counts in regions superior or inferior to the optic nerve. Spider plot shows total cone count per image for individual mice at various distances from the optic nerve head **(A)** and the averaged cone counts per image across treatment groups (*: untreated vs. isotonic buffer: *p* = 0.0224, untreated vs. puncture: *p* = 0.0106) (#: hypertonic vs. untreated: 0.0268, untreated vs isotonic: *p* = 0.0004) **(B)**. Within treatment groups, there is minimal variability in cone distribution across the studied area. Representative 4× immunofluorescent image shows the placement of individual 40× images along the retina and the areas sampled **(C)**.

SUPPLEMENTARY FIGURE S3 **(A–G)** Quantification of cones normalized to untreated fellow eyes demonstrates a trend for higher cone counts in hypertonic buffer-injected eyes. Flat mounts of mouse retinas were collected from untreated eyes and from eyes treated with hypertonic, isotonic, and sham punctured eyes. Sections were stained with PNA to visualize cone outer segments. The number of cones, found in two separate quadrants of an area approximately 350x350 μm², were quantified using ImageJ software and confirmed by masked participants, then averaged. Data were normalized to determine the percentage of cones in the injected eye versus in the uninjected fellow eye. **(A)** The results suggest a trend toward a higher percentage of cones in hypertonic buffer-injected eyes, although this increase was not statistically significant. **(B)** Representative flat mount of a retina from an eye injected with hypertonic buffer. **(C)** Close-up view of the area in one quadrant of the retina shown in **(B)**, highlighting cone outer segments. **(D)** Representation of individual cone counting by ImageJ software. **(E)** Representative flat mount of a retina from an untreated eye. **(F)** Close-up view of the area in one quadrant of the retina shown in **(E)**, highlighting cone outer segments. **(G)** Representation of individual cones labeled by the particle analysis function in ImageJ.
